# High In Vitro Activity of the Novel Pleuromutilin Antibiotic, Lefamulin, on Clinical *Helicobacter pylori* Isolates

**DOI:** 10.3390/antibiotics15080734

**Published:** 2026-07-29

**Authors:** Lyudmila Boyanova, Liliya Yordanova Boyanova, Victor Kamburov, Nayden Kandilarov, Nikolay Katsarov, Raina Gergova, Vasil Svetoslavov Boyanov, Rumyana Markovska

**Affiliations:** 1 Department of Medical Microbiology, Medical University—Sofia, 2 Zdrave Street, 1431 Sofia, Bulgaria; liliya.boianova@medfac.mu-sofia.bg (L.Y.B.); r.gergova@medfac.mu-sofia.bg (R.G.); markovska73@abv.bg (R.M.); 2 Medical Center BalkanMed, 6 Struma Street, 1202 Sofia, Bulgaria; vkamburov@gmail.com; 3 Department of General and Hepatobiliary Pancreatic Surgery, Department of Surgery, Medical Faculty, Medical University of Sofia, 15 Acad. Ivan Geshov Blvd., 1431 Sofia, Bulgaria; naidenk@yahoo.com; 4 Zdraveto Hospital, 4 Maestro Kanev Street, 1618 Sofia, Bulgaria; nkatzarov@yahoo.com

**Keywords:** *Helicobacter pylori*, lefamulin, clarithromycin, levofloxacin, newer antibiotics, susceptibility

## Abstract

**Background**: Antibiotic resistance in *Helicobacter pylori* is steadily increasing, rendering the treatment of related gastroduodenal diseases increasingly difficult. Lefamulin is a new broad-spectrum antibacterial in the pleuromutilin class, acting by suppressing bacterial protein synthesis. It has the advantages of its unique “induced-fit” mechanism, a low frequency of spontaneous mutations, stepwise development of resistance, stability in acidic environments, and potential for additive or synergistic activity when combined with certain other antibiotics against various facultative anaerobes, including multidrug-resistant isolates. **Methods**: We investigated, for the first time to the best of our knowledge, the activity of lefamulin against 91 clinical *H. pylori* isolates from symptomatic adult patients using MIC test strips. **Results**: Overall, lefamulin MICs_50_ and MICs_90_ were 0.25 and 2 mg/L versus 4 and ≥256 mg/L for clarithromycin, and 0.75 and ≥32 mg/L, respectively, for levofloxacin. Lefamulin MICs_50_ and MICs_90_ were 0.5 mg/L and 4 mg/L against the 61 clarithromycin-resistant (MICs, >0.25 mg/L) isolates, 0.25 and 0.75 mg/L against the 40 levofloxacin-resistant (MICs, >1 mg/L) isolates, and 0.38 mg/L and 0.75 mg/L, respectively, against the 28 isolates resistant to both agents. **Conclusions**: Briefly, the new pleuromutilin antibiotic outperformed in vitro both clarithromycin and levofloxacin against *H. pylori* isolates. Its potential usefulness in treating *H. pylori* infections resistant to macrolides and fluoroquinolones, and especially those with dual resistance, justifies further investigation. However, some precautions should also be considered. The use of the novel antibiotic lefamulin may offer benefits for *H. pylori* eradication if our results are confirmed in subsequent studies, including clinical trials.

## 1. Introduction

*Helicobacter pylori* is a causative agent of a widespread, potentially oncogenic and usually lifelong infection if not successfully eradicated. Around 43% of the global human population is infected, and the associated infections can vary from asymptomatic infections to chronic or chronic erosive gastritis, peptic ulcers and tumors such as gastric cancer or MALT lymphoma (mucosa-associated lymphoid tissue lymphoma) [1]. It is important to note that patients infected with *H. pylori* have a 10 to 20% risk of developing peptic ulcer disease and a 1% to ≥2% risk of developing gastric cancer [2].

*H. pylori* resistance to antibiotics can arise in multiple ways, most often by point mutations, but also by efflux mechanisms, biofilm formation, natural transformation and probably transition to viable but non-culturable forms [3,4].

In many countries, *H. pylori* resistance to antibiotics, including macrolides and fluoroquinolones, has been constantly increasing over the years, although in a few countries such as France and Spain, the rise in resistance rates has been stabilized [5]. In the systematic review and meta-analysis of Khoshnood et al. [6], encompassing 89 articles, clarithromycin resistance in *H. pylori* was highly variable worldwide, ranging from 7% in the USA to >93% in Australia. In the meta-analysis of Yu et al. [7], in 2013–2023, the primary resistance to clarithromycin was from 16.0% in American countries to >28.0% in Asia. Primary levofloxacin resistance rates in 2013–2023 also widely varied, ranging from 5.2 to 52.8% in America and from 0.0 to around 50.0% in Africa [7].

In Bulgaria, the overall resistance frequency in *H. pylori* increased for all antibiotics tested except for tetracycline. Amoxicillin resistance rates rose significantly between 2007 and 2014 and 2015–2021, while metronidazole, clarithromycin, and levofloxacin resistance rates rose significantly from 2010 to 2015 to 2016–2022 [5]. Therefore, data on the activity of new antibiotics can help improve the treatment of infections if the in vitro results are subsequently confirmed clinically.

Genes/sequences conferring *H. pylori* resistance were often the A2143G, A2142G, and A2142C mutations in 23S rRNA, but also the *hefA* efflux pump gene or *rpl22* and *infB* gene modifications for clarithromycin, and point mutations in the quinolone resistance-determining region of the gyrase-encoding *gyrA* and *gyrB* genes for levofloxacin [8,9,10].

To overcome the problems associated with *H. pylori* antibiotic resistance, different approaches were employed, such as the use of bismuth and non-bismuth quadruple regimens, new acid-suppressive agents such as vonoprazan (a potassium-competitive acid blocker), an increase in the length of triple therapy, tailored therapy, etc. [7,11,12]. Another approach is the search for new combination regimens or newer antibiotics that demonstrate advantages over those commonly used to eradicate the infection.

The new antibiotic in the pleuromutilin class, lefamulin, is a recent semisynthetic and broad-spectrum antibacterial drug that acts by inhibiting the synthesis and growth of bacterial proteins, targeting the site of the protein folding (domain V) of the 23S rRNA in the bacterial 50S subunit [13].

Both the Food and Drug Administration (in 2019) and the European Medicines Agency (in 2020) have authorized the medication for the treatment of bacterial pneumonia obtained in the community. Lefamulin was effective against Gram-positive microbes such as *Streptococcus pneumoniae*, *Enterococcus faecium* and *Staphylococcus aureus*, and some Gram-negative bacteria such as *Haemophilus*, *Legionella*, *Mycoplasma*, and *Chlamydia* spp. [14]. The pleuromutilin antibiotic is available in oral and parenteral preparations. Taylor et al. [13] emphasized that lefamulin is a promising alternative to macrolides and fluoroquinolones for treating the frequent causative agents of infections of the respiratory tract.

To the best of our knowledge, there have been no publications to date regarding the activity of lefamulin against *H. pylori*.

The aim of this study was to investigate the in vitro susceptibility of clinical *H. pylori* isolates to lefamulin, compared to that of the macrolide clarithromycin, frequently used for eradication of the infection, and the fluoroquinolone antibiotic levofloxacin, used in second-line or rescue regimens. Lefamulin was chosen because there is currently a lack of data on its efficacy against *H. pylori*, although it is necessary to investigate many potential options for improving the suboptimal eradication regimens for the associated widespread and potentially oncogenic infection.

## 2. Results

Among the 91 *H. pylori* isolates in the present study, resistance rates to clarithromycin, levofloxacin and both antibiotics were 67.0%, 44.0%, and 30.8%, respectively.

At 0.25 mg/L (*H. pylori* susceptibility/resistance breakpoint for clarithromycin according to the EUCAST, version 16, 2026), more isolates were susceptible to lefamulin (47, 51.6%) compared to clarithromycin (30, 33.0%, *p* = 0.016), [15]. At 1 mg/L (*H. pylori* susceptibility/resistance breakpoint for levofloxacin according to the EUCAST recommendations), more isolates were susceptible to lefamulin (77, 84.6%) compared to levofloxacin (51, 56.0%, *p* = 0.0001), [15]. The results are presented in more detail in Table 1.

For lefamulin, the MICs_50_, MICs_90_, and MIC ranges were 0.25, 2, and 0.016–≥256 mg/L; for clarithromycin, they were 4, ≥256, and 0.016–≥256 mg/L; and for levofloxacin, the values were 0.75, ≥32, and 0.002–≥32 mg/L. The MIC values are shown in detail in Table 2.

Against the 61 clarithromycin-resistant isolates (MICs, >0.25 mg/L), lefamulin MICs_50_ and MICs_90_ were 0.5 mg/L and 4 mg/L. The lefamulin MICs_50_ and MICs_90_ against the 40 isolates resistant to levofloxacin (MICs, >1 mg/L) were 0.25 mg/L and 0.75 mg/L, respectively. Against the isolates resistant to both clarithromycin and levofloxacin, the MICs_50_ and MICs_90_ of the novel pleuromutilin were 0.38 mg/L and 0.75 mg/L, respectively. 

## 3. Discussion

Although we did not have complete data on the patients’ previous therapy, currently, more than four decades after *H. pylori* discovery, most symptomatic patients have undergone one or several eradication attempts, which may explain the high rates of resistance to macrolides and fluoroquinolones in the present study. In our work, clarithromycin and levofloxacin resistance rates were 1.4- and 1.8-fold higher, respectively, than those in the study of Bujanda et al. [16], reporting resistance to clarithromycin in 49% and to quinolones in 24% among non-naive patients in Europe. The difference can be due to the high J01F (macrolide, lincosamide and streptogramin) consumption (5.5 defined daily doses, DDDs per 1000 inhabitants per day) in Bulgaria in 2021, compared to that of other European countries [5]. Likewise, in our country, the high levofloxacin resistance may be linked to the high J01M (quinolone) use (3.9 DDDs per 1000 inhabitants per day) compared to that in other European countries such as France (1.0 DDD) [5]. Moreover, unlike many other countries in Europe, Bulgaria exhibited an increasing trend in the use of systemic antibiotics in the community and hospitals in 2019–2023 [17].

In the present study, metronidazole resistance was also high (63.7%, 68/91 isolates). However, we did not use this agent as a comparator, because *H. pylori* eradication in the presence of metronidazole resistance can be obtained by increasing the doses of either metronidazole or proton pump inhibitors, by adding bismuth salts, or by prolonging the length of eradication therapy [18,19,20,21]. Recently, Bujanda et al. [16] reported in a Hp-EuReg multicenter study that regardless of metronidazole resistance, the quadruple regimen with a proton pump inhibitor and Pylera capsules, containing bismuth subcitrate potassium, metronidazole, and tetracycline, can reach a high (>90%) eradication success.

Lefamulin is a new antibacterial in the pleuromutilin class that uses a specifically induced mechanism of tight-fit attachment to the A and P sites of the 50S ribosomal subunit to suppress bacterial protein synthesis by interfering with peptidyl transferase, which in turn prevents the creation of peptide bonds and chain elongation [22]. This antibiotic has a tricyclic core, containing a 14-carbon ring that is important for antibacterial activity, a ketone group, and a C14 side chain that counteracts the development of resistance [22,23].

In the present study, we compared the MIC values of the isolates for lefamulin to those for clarithromycin and levofloxacin, using the EUCAST resistance breakpoints available for these antimicrobial agents [15], since there are no breakpoints for the new antibiotic against *H. pylori.*

However, Kozhokar et al. [14] emphasize that the new pleuromutilin should not be used in patients with a prolonged QT interval or ventricular arrhythmias, nor in those taking macrolides such as erythromycin or fluoroquinolones such as moxifloxacin and levofloxacin, which can prolong the QT interval. Although no dose adjustment of lefamulin is necessary in cases of renal impairment, the agent should be avoided in patients with severe liver disease or renal failure requiring dialysis [14,24]. It should also be noted that, like many other broad-spectrum antibiotics, lefamulin may cause *Clostridioides difficile* infection [14]. Therefore, in our opinion, it should always be prescribed with a recommendation for simultaneous use of probiotics.

Lefamulin resistance rates against some facultatively anaerobic species were very low. In the recent review article of Falagas et al. [23], encompassing 11 publications, the resistance rates of lefamulin ranged from 0 to 2.6% for *S. pneumoniae* (at >0.5 mg/L), from 0 to 2.4% for *Haemophilus influenzae* (at >2 mg/L), and from 0 to 4.3% for *S. aureus* isolates (at >0.25 mg/L) according to the EUCAST (Version 15.0) and CLSI (M100) breakpoints.

The distinct mode of action, the low (≤10^−9^) spontaneous mutation rates, and the gradual (stepwise) development of resistance indicated a minimal likelihood of resistance emerging during therapy [23,25]. Possible causes of resistance to lefamulin can be some 23S rRNA mutations, the genes *rplC* and *rplD* encoding the ribosomal proteins L3 and L4, the *vga*(A) transporter gene, and the *cfr* gene encoding the Cfr methyltransferase and multidrug resistance [23,25]. Even though there is currently no breakpoint for *H. pylori* resistance to lefamulin, future research should focus on the effects of various genetic mechanisms linked to elevated MICs of the agent against *H. pylori*, including different point mutations in the ribosomal proteins and efflux pump overexpression.

Another strong advantage of lefamulin is that it did not exhibit cross-resistance with macrolides, as reported for erythromycin-resistant isolates of *S. pneumoniae* in the study by Mendes et al. [26], nor with macrolide-resistant *Mycoplasma pneumoniae* mutants in the study by Waites et al. [27], nor with azithromycin-resistant *Neisseria gonorrhoeae* [28]. Furthermore, the pleuromutilin antibiotic exhibits stability in an acidic environment and may therefore be active in the gastric mucosa [29].

Against the 251 gonococcal isolates in the study of Jacobsson et al. [28], MICs_90_ of lefamulin were lower than those of azithromycin, tetracycline and ciprofloxacin. In this study, the MICs_50_ and MICs_90_ of lefamulin were 0.25 and 1 mg/L, compared to 0.5 and 16 mg/L for azithromycin, 2 and 16 mg/L for tetracycline, and 0.25 and >32 mg/L for ciprofloxacin, respectively [28].

The pleuromutilin antibiotic was active in the presence of certain efflux pumps. Although the efflux pump MtrCDE affected the susceptibility to lefamulin in *N. gonorrhoeae* isolates, MacAB and NorM efflux pumps did not influence its activity [28].

Combinations of lefamulin with other antibiotics have been evaluated in recent studies. The effect of lefamulin in combination with doxycycline and rifampin was synergistic in most (>85 to 97%) of the 34 *Enterococcus faecium* isolates tested in the study of Min et al. [30]. In another study [31], there was not any antagonistic effects in a checkerboard assessment of the combination of lefamulin and doxycycline for 54 *Mycoplasma genitalium* isolates but there was an additive effect in some isolates.

Therefore, lefamulin may be a promising antibiotic for treating antibiotic-resistant bacteria, possibly including *H. pylori*, but further in vitro studies in different countries and clinical trials are needed to confirm its efficacy. If confirmed, the present results could be beneficial not only for Bulgaria but also for neighboring countries such as Greece, where *H. pylori* resistance rates were up to 37% for metronidazole and 27% for clarithromycin, and secondary resistance rates in 2013 were 50.6% for metronidazole and 72.3% for clarithromycin [32].

## 4. Limitations of the Study

The present study has several limitations, such as the sample size of only 91 clinical *H. pylori* isolates, a study conducted in a single laboratory, and the lack of molecular characterization of isolates.

## 5. Materials and Methods

### 5.1. Ethics Statement 

This study was conducted on bacterial isolates according to the standard susceptibility testing protocol for *H. pylori* isolates in the laboratory’s routine diagnostic work. The work did not include any procedures beyond those routinely performed for diagnostic purposes. Informed written consent was obtained from all patients by the clinicians in the gastroenterology units.

### 5.2. Patients and H. pylori Isolation

We examined the activity of lefamulin in comparison to clarithromycin and levofloxacin as comparators against 91 clinical *H. pylori* isolates from symptomatic adult patients from the end of 2024 (4 isolates) and 2025 (57 isolates) to 2026 (30 isolates).

The patients included 55 women and 36 men. All patients were adults and based on the available data on the exact age of 81 of them, the mean age was 50 years, ranging from 21 to 81 years.

Among all patients, 70 had chronic gastritis, five suffered from peptic ulcers, seven had reflux esophagitis/gastroesophageal reflux disease (GERD), eight were diagnosed with both erosive gastritis and reflux esophagitis/GERD, and one patient had gastroenteroanastomosis. Detailed data are presented in Table 3.

*H. pylori* isolation and identification were performed as reported in our prior study [33] and in accordance with well-established expert recommendations [34]. Briefly, *H. pylori* isolation was made from gastric biopsy samples obtained by gastroenterologists in their endoscopy suites and sent to the laboratory. For cultivation, as necessary for optimal *H. pylori* isolation, we used two types of culture media: non-selective Mueller–Hinton agar with 5% defibrinated horse blood and selective agar with Dent’s supplement for *H. pylori*. The agar plates were incubated in a microaerophilic atmosphere using CampyGen sachets at 37 °C for 3 to 10 days. All necessary supplies were purchased from Thermo Fisher Oxoid (Basingstoke, UK). Identification of the isolates was based on their characteristic spiral-shaped morphology in microscopic preparations from both gastric specimens and pure cultures, as well as on the absence of growth under aerobic conditions and positive results for the tests for catalase, oxidase, and the rapid urease test specific to these bacteria.

### 5.3. Susceptibility Testing

For susceptibility testing, we used Mueller–Hinton agar (Thermo Scientific Oxoid, Basingstoke, Hampshire, UK) with 5% horse blood (Thermo Scientific Oxoid), *H. pylori* inocula corresponding to McFarland turbidity standards of 2 to 4, as recommended, and MIC test strips (Liofilchem, Roseto degli Abruzzi, Italy). The antibiotic-susceptible *H. pylori* CCUG 18742 isolate was used for quality control as in the review article of Mégraud and Lehours [34].

The plates were incubated in a microaerophilic atmosphere with CampyGen gas generator envelopes (Thermo Scientific Oxoid) at 37 °C for 2 to 3 days.

Resistance breakpoints were >0.25 mg/L for clarithromycin and >1 mg/L for levofloxacin according to the EUCAST breakpoints, Version 16.1, 2026 [15]. There are no resistance breakpoints for lefamulin against *H. pylori*.

### 5.4. Statistical Analysis

For statistical analysis, the chi-square with Fisher’s exact test was used and *p* values < 0.5 were considered significant.

## 6. Conclusions

In conclusion, it is noteworthy that the novel pleuromutilin antibiotic has shown the advantages of its unique “induced-fit mechanism” of action, a low rate of spontaneous mutations, a slow (gradual) development of resistance, stability in an acidic environment, and potential for additive or synergistic activity when combined with certain other antibiotics against various Gram-positive and Gram-negative bacteria, including multidrug-resistant isolates. In our study, lefamulin performed better against *H. pylori* isolates in vitro than both levofloxacin and clarithromycin, including against isolates with single or dual resistance. The MIC_50_ values found for lefamulin were 16-fold lower than those for clarithromycin and 3-fold lower than those for levofloxacin. The MIC_90_ values for the new antibiotic were 128-fold lower than those for clarithromycin and 16-fold lower than those for levofloxacin. Further research on this novel antibiotic is warranted due to its potential utility in treating *H. pylori* infections resistant to macrolides and fluoroquinolones, particularly those with dual resistance to the antibiotics from both classes employed in eradication regimens. If our results are confirmed in further studies, including clinical trials, the introduction of the novel antibiotic lefamulin could help improve treatment outcomes for the widespread *H. pylori* infection, which has become increasingly difficult to eradicate.

## Figures and Tables

**Table 1:** Susceptibility patterns of lefamulin, clarithromycin, and levofloxacin at the EUCAST breakpoints of EUCAST, v. 16.1 for clarithromycin (0.25 mg/L) and levofloxacin (1 mg/L) [15].

Antibiotic (Class)/ Breakpoint	Isolates (No.)	Lefamulin (Pleuromutilin)	Clarithromycin (Macrolide)	Levofloxacin (Fluoroquinolone)
EUCAST breakpoints		NA	0.25 mg/L	1 mg/L
At 0.25 mg/L	Susceptible	**47 (51.6%)**	**30 (33.0%)**	33 (36.3%)
	Non-susceptible	**44 (48.4%)**	**61 (67.0%)**	58 (63.7%)
At 1 mg/L	Susceptible	**77 (84.6%)**	43 (47.3%)	**51 (56.0%)**
	Non-susceptible	**14 (15.4%)**	48 (52.7%)	**40 (44.0%)**

NA—not available; the data regarding EUCAST breakpoints [15] are presented in bold.

**Table 2:** Minimal inhibitory concentrations (MICs) of lefamulin against clinical *H. pylori* isolates.

*H. pylori* Isolates	Antibiotic	No. of Isolates Tested	MIC_50_ (mg/L)	MIC_90_ (mg/L)	MIC Range (mg/L)
Total	Clarithromycin	91	4	≥256	0.016–≥256
Total	Lefamulin	91	0.25	2	0.016–≥256
Total	Levofloxacin	91	0.75	≥32	0.002–≥32
Clarithromycin resistant subgroup	Lefamulin	61	0.5	4	0.016–≥256
Levofloxacin resistant subgroup	Lefamulin	40	0.25	0.75	0.016–32
Dual resistant subgroup	Lefamulin	28	0.38	0.75	0.016–32

**Table 3:** Symptomatic adult patients included in the study.

Groups According to the	Groups	No. of Patients
Sex	Men	36
	Women	55
Age *	21–64 years	75
	65–81 years	6
	Non-specified age of adult patients	10
Diagnosis	Chronic gastritis	45
	Chronic erosive gastritis	24
	Chronic atrophic gastritis	1
	Gastric ulcer	3
	Duodenal ulcer	2
	Reflux esophagitis/GERD **	7
	Erosive gastritis and reflux esophagitis/GERD	8
	Gastroenteroanastomosis	1
Total		91

* For the patients with specified age: mean age 50.0 years, range 21–81 years; ** GERD—Gastroesophageal reflux disease.

## Data Availability

All datasets generated or analyzed during the study are included in the manuscript.

## References

[1] Li Y., Choi H., Leung K., Jiang F., Graham D.Y., Leung W.K. (2023). Global prevalence of *Helicobacter pylori* infection between 1980 and 2022: A systematic review and meta-analysis. Lancet Gastroenterol. Hepatol..

[2] Sánchez E.D.T., Nieto M.M., Alvarez G.E.G., Montaño R.R., Alarcón-Sánchez M.A., Heboyan A., Maldonado A.F.G., Hernández J.J.V., Martínez S.M.L. (2026). *Helicobacter pylori* in oral and gastric pathologies: A narrative review of potential bidirectional pathogenic interactions. Ann. Med..

[3] Tshibangu-Kabamba E., Yamaoka Y. (2021). *Helicobacter pylori* infection and antibiotic resistance—From biology to clinical implications. Nat. Rev. Gastroenterol. Hepatol..

[4] Elbaiomy R.G., Luo X., Guo R., Deng S., Du M., El-Sappah A.H., Bakeer M., Azzam M.M., Elolimy A.A., Madkour M. (2025). Antibiotic resistance in *Helicobacter pylori*: A genetic and physiological perspective. Gut Pathog..

[5] Boyanova L., Hadzhiyski P., Gergova R., Markovska R. (2023). Evolution of *Helicobacter pylori* resistance to antibiotics: A topic of increasing concern. Antibiotics.

[6] Khoshnood S., Pakzad R., Kaviar V.H., Hashemian M., Karamollahi S., Sadeghifard N., Heidarizadeh H., Moradi M., Maleki A., Heidary M. (2023). Global estimate of clarithromycin resistance in clinical isolates of *Helicobacter pylori*: A systematic review and meta-analysis. Clin. Lab..

[7] Yu Y., Xue J., Lin F., Liu D., Zhang W., Ru S., Jiang F. (2024). Global primary antibiotic resistance rate of *Helicobacter pylori* in recent 10 years: A systematic review and meta-analysis. Helicobacter.

[8] Álvarez-Aldana A., Beltrán-Angarita L., Guaca-González Y.M., Velandia-López M.A., Boyanova L. (2026). Levofloxacin and rifampin resistance in *Helicobacter pylori* isolates from Central-Western Colombia: Role of *gyrA* mutations in fluoroquinolone resistance. Antibiotics.

[9] Hasanuzzaman M., Bang C.S., Gong E.J. (2024). Antibiotic resistance of *Helicobacter pylori*: Mechanisms and clinical implications. J. Korean Med. Sci..

[10] Villegas M., Ortega C., Gómez K., Cerda A., Sepúlveda R., Lara C., Bustamante L., Garcia D., Coppelli L., Hofmann E. (2025). High Prevalence of *hefA* efflux pump overexpression in isolates of *Helicobacter pylori* resistant to clarithromycin. Antibiotics.

[11] Roszczenko-Jasińska P., Wojtyś M.I., Jagusztyn-Krynicka E.K. (2020). *Helicobacter pylori* treatment in the post-antibiotics era-searching for new drug targets. Appl. Microbiol. Biotechnol..

[12] Rocha G.R., Lemos F.F.B., Silva L.G.O., Luz M.S., Santos G.L.C., Pinheiro S.L.R., Calmon M.S., de Melo F.F. (2025). Overcoming antibiotic-resistant *Helicobacter pylori* infection: Current challenges and emerging approaches. World J. Gastroenterol..

[13] Taylor R.M., Karlowsky J.A., Baxter M.R., Adam H.J., Walkty A., Lagacé-Wiens P., Zhanel G.G. (2021). In vitro susceptibility of common bacterial pathogens causing respiratory tract infections in Canada to lefamulin, a new pleuromutilin. J. Assoc. Med. Microbiol. Infect. Dis. Can..

[14] Kozhokar L., Levien T.L., Baker D.E. (2022). Lefamulin. Hosp. Pharm..

[15] The European Committee on Antimicrobial Susceptibility Testing Breakpoint Tables for Interpretation of MICs and Zone Diameters. Version 16.1, 2026. https://www.eucast.org.

[16] Bujanda L., Nyssen O.P., Ramos J., Bordin D.S., Tepes B., Perez-Aisa A., Pavoni M., Castro-Fernandez M., Lerang F., Leja M. (2024). Effectiveness of *Helicobacter pylori* treatments according to antibiotic resistance. Am. J. Gastroenterol..

[17] European Centre for Disease Prevention and Control (2024). Antimicrobial Consumption in the EU/EEA (ESAC-Net)—Annual Epidemiological Report 2023.

[18] van Zwet A.A., Thijs J.C., de Graaf B. (1995). Explanations for high rates of eradication with triple therapy using metronidazole in patients harboring metronidazole-resistant *Helicobacter pylori* strains. Antimicrob. Agents Chemother..

[19] Andersen L.P., Colding H., Kristiansen J.E. (2000). Potentiation of the action of metronidazole on *Helicobacter pylori* by omeprazole and bismuth subcitrate. Int. J. Antimicrob. Agents..

[20] Vakil N., Megraud F. (2007). Eradication therapy for *Helicobacter pylori*. Gastroenterology.

[21] Liou J.M., Lee Y.C., Wu M.S., Taiwan Gastrointestinal Disease and Helicobacter Consortium (2022). Treatment of refractory *Helicobacter pylori* infection-tailored or empirical therapy. Gut Liver.

[22] Mercuro N.J., Veve M.P. (2020). Clinical utility of lefamulin: If not now, when?. Curr. Infect. Dis. Rep..

[23] Falagas M.E., Fanariotis G., Romanos L.T., Katsikas K.M., Kakoullis S.A. (2026). Resistance to Lefamulin: An Evaluation of Data from In Vitro Antimicrobial Susceptibility Studies. Antibiotics.

[24] Wang H., Charles C.V. (2020). A Review of newly approved antibiotic treatment for community-acquired bacterial pneumonia: Lefamulin. Sr. Care Pharm..

[25] Zhanel G.G., Deng C., Zelenitsky S., Lawrence C.K., Adam H.J., Golden A., Berry L., Schweizer F., Zhanel M.A., Irfan N. (2021). Lefamulin: A novel oral and intravenous pleuromutilin for the treatment of community-acquired bacterial pneumonia. Drugs.

[26] Mendes R.E., Farrell D.J., Flamm R.K., Talbot G.H., Ivezic-Schoenfeld Z., Paukner S., Sader H.S. (2016). In vitro activity of lefamulin tested against *Streptococcus pneumoniae* with defined serotypes, including multidrug-resistant isolates causing lower respiratory tract infections in the United States. Antimicrob. Agents Chemother..

[27] Waites K.B., Crabb D.M., Duffy L.B., Jensen J.S., Liu Y., Paukner S. (2017). In vitro activities of lefamulin and other antimicrobial agents against macrolide-susceptible and macrolide-resistant *Mycoplasma pneumoniae* from the United States, Europe, and China. Antimicrob. Agents Chemother..

[28] Jacobsson S., Paukner S., Golparian D., Jensen J.S., Unemo M. (2017). in vitro activity of the novel pleuromutilin lefamulin (BC-3781) and effect of efflux pump inactivation on multidrug-resistant and extensively drug-resistant *Neisseria gonorrhoeae*. Antimicrob. Agents Chemother..

[29] Usman M., Abbas T. (2023). Development and validation of a stability-indicating RP-HPLC method for estimation of lefamulin in pure and pharmaceutical drug products. Future J. Pharm. Sci..

[30] Min Y.H., Kim Y.U., Park M.C. (2024). In vitro synergistic effect of lefamulin with doxycycline, rifampin, and quinupristin/dalfopristin against enterococci. Microorganisms.

[31] Salado-Rasmussen K., Nørgaard C., Pedersen T.R., Paukner S., Jensen J.S. (2025). In vitro test of the novel antibiotic lefamulin alone and in combination with doxycycline against *Mycoplasma genitalium*. Antimicrob. Agents Chemother..

[32] Georgopoulos S.D., Michopoulos S., Rokkas T., Apostolopoulos P., Giamarellos E., Kamberoglou D., Mentis A., Triantafyllou K. (2020). Hellenic consensus on *Helicobacter pylori* infection. Ann. Gastroenterol..

[33] Boyanova L., Hadzhiyski P. (2020). *Helicobacter pylori* infection is associated with anemia, weight loss or both conditions among Bulgarian children. Acta Microbiol. Immunol. Hung..

[34] Mégraud F., Lehours P. (2007). *Helicobacter pylori* detection and antimicrobial susceptibility testing. Clin. Microbiol. Rev..

